# *Lactobacillus sakei* S1 Improves Colitis Induced by 2,4,6-Trinitrobenzene Sulfonic Acid by the Inhibition of NF-κB Signaling in Mice

**DOI:** 10.4014/jmb.1907.07050

**Published:** 2019-10-13

**Authors:** Se-Eun Jang, Sung-Won Min

**Affiliations:** 1 Department of Food and Nutrition, Eulji University, Seongnam 335, Republic of Korea; 2 SG Medical, Seoul, Republic of Korea

**Keywords:** *Lactobacillus sakei*, probiotics, colitis, 2, 4, 6-trinitrobenzene sulfonic acid (TNBS), NF-κB

## Abstract

*Lactobacillus sakei* S1 strongly inhibits the expression of interleukin (IL)-6 and IL-1β in lipopolysaccharide-induced peritoneal macrophages by a mechanism for which lactic acid bacteria from kimchi that inhibit tumor necrosis factor-alpha (TNF-α) were isolated. Therefore, we further evaluated the protective effect of this strain on the colitis mouse model induced by 2,4,6-trinitrobenzene sulfonic acid (TNBS). TNBS significantly elevated myeloperoxidase (MPO) expression, macroscopic scores, and colon shortening. Oral *L. sakei* S1 administration resulted in reduction of TNBS-induced loss in body weight, colon shortening, MPO activity, expression of cyclooxygenase (COX)-2, inducible nitric oxide synthase (iNOS) and nuclear factor-kappa B (NF-κB). *L. sakei* S1 inhibited the expression of inflammatory cytokines IL-1β, IL-6 and TNF-α, induced by TNBS, but enhanced IL-10 expression. *L. sakei* S1 showed resistance to artificial digestive juices and adherence to intestinal epithelial Caco-2 cells. Thus, *L. sakei* S1 may inhibit the NF-κB pathway and be used in functional food to treat colitis.

## Introduction

Either progressive or chronic remittent inflammatory conditions are the main characteristics of inflammatory bowel disease (IBD), a group of conditions that include Crohn’s disease and ulcerative colitis and cause injury to the colonic mucosa or even the complete gastrointestinal tract, leading to recurrent diarrhea and abdominal pain [1, 2]. Although a clear pathogenesis of IBD has not been established yet, accumulating reports indicate the impaired regulation of immune response of the intestine towards the endotoxins present in intestinal microflora and similar environmental antigens [3]. The human gut microbiota consists of 10 to 100 trillion microorganisms [3]. Among them, more than 1,000 gut bacterial species such as *Enterobacteriaceae* are closely related to the onset and persistence of ulcerative colitis [4]. Gram-negative bacteria, including *Enterobacteriaceae*, generate bacterial endotoxins such as lipopolysaccharides (LPS). LPS originating from gut bacteria contribute to the development of metabolic as well as inflammatory disorders [3]. LPS activates NF-κB signaling in macrophages, and this leads to enhanced production of different inflammatory cytokines, such as interleukin (IL)-1β, tumor necrosis factor-alpha (TNF-α), etc. [5]. Inflammation is caused by host immune responses to pathogenic or tissue damage and is mediated by cytokines. TNF-α and IL-1β are the main pro-inflammatory cytokines that cause inflammation [6]. LPS activation of macrophages is the major source of the formation and release of these pro-inflammatory cytokines, which promote the inflammatory response [7]. NF-κB transcription factor is involved in the transcriptional control of the expression of TNF-α and IL-1β [6, 7]. Thus, the focus of most recent research has been to identify a dietary component that can inhibit the LPS-induced NF-κB signaling pathway to ameliorate IBD [8].

Lactic acid bacteria (LAB) are considered to be beneficial and non-pathogenic microorganisms in the digestive tract [9]; they exhibit anti-obesity effects [10], mitigate infectious and inflammatory diseases [11], and have anti-colic effects [12]. For instance, in mice with dextran sulfate sodium (DSS)-induced colitis, *Lactobacillus gasseri* blocks the biosynthesis of inflammatory cytokines [13]. Furthermore, *Lactobacillus casei*, *Bifidobacterium lactis*, *Lactobacillus plantarum* and *Bifidobacterium longum* also exhibit anti-inflammatory properties in the colitis model [14, 15]. Although several strains of LAB that have anti-colitis effects have been reported, studies on the anti-colitis effects of the new strain of lactic acid bacteria may be helpful in the treatment of socially severe and refractory colitis.

Thus, employing peritoneal macrophages that are stimulated with LPS, the inhibition of TNF-α expression by LAB from kimchi was assessed. Our results revealed the anti-colitis effect of S1, which was identified as *L. sakei* through genetic analyses.

## Materials and Methods

### Materials

TNBS, sodium thioglycolate, LPS from *Escherichia coli* O111:B4, and Roswell Park Memorial Institute (RPMI) medium were obtained from Sigma-Aldrich (USA). Radio immunoprecipitation assay (RIPA) buffer was obtained from Abcam (UK). Enzyme-linked immunosorbent assay (ELISA) kits for IL-6, IL-1β, and TNF-α were purchased from eBioscience (USA). Antibodies against p-p65, p65, cyclooxygenase (COX)-2, inducible NO synthase (iNOS), and β-actin were procured from Cell Signaling Technology (USA) and Abcam (USA). A Pierce enhanced chemiluminescence (ECL) western blotting substrate was obtained from Thermo Fisher Scientific (USA). The Gram staining kit was obtained from BioMerieux (France).

### Isolation and Culture of *L. sakei* S1

Forty LAB strains isolated from a Korean traditional fermented food (Chinese cabbage kimchi), were cultured in MRS broth (BD, USA). Identification of the isolated LAB strains was done by 16S ribosomal DNA sequencing and Gram staining, by using previously published procedures [3]. An assay was performed to determine the anti-inflammatory activity of LAB in macrophages. LAB were grown in MRS broth under anaerobic conditions at 37°C. Bacterial cultures were then centrifuged for 20 min at 5,000 ×*g* and the bacterial pellets were washed with saline. For in vitro assay, the pelleted bacteria were inactivated by being kept at 72°C for 30 min, after their suspension in 1 ml of saline (PBS). For in vivo assay, the LAB strain *L. sakei* S1 was grown in MRS broth until the optical density at 600 nm was between 1 and 2, followed by centrifugation (5,000 ×*g* for 20 min), and washing of the pellet with saline. The collected bacterial cells were suspended in 1%glucose at a density of either 1 × 10^8^ CFU or 1 × 10^9^ CFU, for oral administration in mice.

### Survival Rate of *L. sakei* S1 on Gastric and Intestinal Juice

To determine the survival rate in artificial gastric juice and intestinal juice, *L. sakei* S1 was first inoculated into artificial gastric juice, cultured at 37°C for 3 h and centrifuged to recover the cells [16]. The recovered bacteria were mixed with the artificial intestinal fluid and incubated for 3 h at 37°C. The number of surviving *L. sakei* S1 was measured by plating on MRS agar. Preparation of artificial gastric juice involved mixing of MRS broth, pH 2.5, adjusted with 1 N HCl, and pepsin (1 mg/ml). The artificial intestinal fluid was adjusted to pH 6.8 with Gram broth containing musin (0.1%), pancreatin (0.04%), bile salt (0.2%), trypsin (0.04%), and NaCl (0.85%).

### The Ability of *L. sakei* S1 to Attach to Intestinal Epithelial Cells

In order to measure the adherence of isolated LAB strains to intestinal epithelial cells, Caco-2 cells were used. Caco-2 cells obtained from Korean Cell Line Bank (KCLB) were grown in DMEM (Dulbecco’s modified Eagle’s medium) supplemented with 10% (v/v) fetal bovine serum (FBS, Sigma-Aldrich), 1 mM sodium pyruvate, 2 mM L-glutamine, 0.1 mg/ml streptomycin and 100 U/ml penicillin, in 5% CO_2_ incubator at 37°C. The Caco-2 cells were then harvested and washed with antibiotic-free DMEM and cultured in 6-well culture plates in antibiotic-free DMEM supplemented with FBS, by adjusting the number of Caco-2 cells to 1.0 × 10^5^ cells/ml to form the monolayer. After inoculation, the cells were incubated in 5% CO_2_ incubator for 2 h at 37°C.

The culture of the *L. sakei* S1 in the MRS liquid medium at 37°C for 24 h was centrifuged to collect only the bacteria, washed with PBS, pH 7.0, and the number of *L. sakei* S1 bacterial cells in the DMEM medium was adjusted to 1.0 × 10^7^ CFU/ml. Caco-2 cells were preincubated with a *L. sakei* S1 suspension and cultured at 37°C for 2 h. Non-adherent cells were discarded, and the cells were desensitized with trypsin-EDTA solution to obtain PBS (pH 7.0) and cultured on MRS agar plates to determine the number of *L. sakei* S1 [16].

Cells were washed twice with PBS (pH 7.0) and centrifuged (7,000 ×*g*, 10 min) at 37°C for 24 h in MRS broth to measure autoaggregation. The cell suspension (2 ml) was vortexed vigorously for approximately 10 s and incubated at 37°C for 2 h. The supernatant (1 ml) was collected, and the absorbance at 600 nm was measured. The self-cohesive force (%) was determined by substituting the equation [(1 − absorbance after incubation for 2 h/absorbance before incubation) × 100] [17].

### Animals

Five-week-old male ICR mice (weighing 26–28 g) were supplied from Orient Bio Inc. (Korea). Mice were housed and maintained at 20 ± 5°C and 12 h dark/light cycles, at six mice per cage, with full access to a diet of standard laboratory chow and water. All animal experiments were approved by the Committee for the Care and Use of Laboratory Animals at Eulji University and performed in accordance with the guidelines of the Eulji University Institutional Animals Care and Usage Committee (IACUC) (Approval No. EUIACUC 18-06).

### Preparation of Macrophages

Male ICR mice were given thioglycolate injections (2 ml of 4%solution (w/v); intraperitoneally) and were sacrificed 4 days after the injection [18]. RPMI 1640 (5 ml) was used to flush the peritoneal cavity to collect the peritoneal fluid, which was centrifuged for 5 min at 300 ×*g*. Approximately 1 × 10^6^ cells were seeded in culture plates, incubated in RPMI 1640 containing 10%FBS and 1% antibiotic-antimycotic at 37°C overnight, and washed twice. The cells attached to the plates were employed as macrophages. To determine the levels of pro-inflammatory cytokines, LPS (100 ng/ml) was added to the cultured peritoneal macrophages (1 × ^6^ cells/well), either with or without a varying number of *L. sakei* S1 (1 × 10^3^, 1 × 10^4^, or 1 × 10^5^ CFU/well) for 24 h.

### Induction and Evaluation of Colitis in Mice

For in vivo experiment, mice were separated into 5 experimental groups; (1) a vehicle only treated normal control group; (2) a TNBS only given control group; (3, 4) two separate groups treated with TNBS and *L. sakei* S1 at 1 × 10^8^ or 1 × 10^9^ CFU/mouse, respectively; (5) a positive control group of mice treated with sulfasalazine (50 mg/kg) and TNBS.

Induction of colitis was done by the intrarectal injection of a 2.5% (w/v) TNBS solution (100 μl) in 50% (v/v) ethanol into the colon of the mice [18]. For intrarectal injection of TNBS solution, the needle was introduced about 3.5–4 cm proximal to the anus area. In order to achieve even distribution of TNBS solution throughout colon, mice were held vertically for 30 sec following TNBS injection. *L. sakei* S1 (1 × 10^8^ CFU/mouse or 1 × 10^9^ CFU/mouse) or sulfasalazine (50 mg/kg) was orally administered once a day for 3 days after TNBS treatment. Mice were sacrificed 18 h after the final administration of *L. sakei* S1 or sulfasalazine. The colon was removed rapidly, and washed gently with saline after opening it longitudinally. Assessment of colitis by macroscopic score was as follows: 0, absence of ulcer or inflammation; 1, no ulcers or local hyperemia; 2, ulceration in the absence of hyperemia; 3, one site only of inflammation and ulceration; 4, two or more sites of inflammation and ulceration; 5, ulceration spreading >2 cm (Lim *et al*., 2016). Colonic tissues were stored at −80°C until experiments were completed, and some tissues were stored in 4%PFA for tissue staining.

### Myeloperoxidase (MPO) Activity Assay

Activity of MPO was assayed according to previously described procedures by Lim *et al*. [18]. The colon tissues were homogenized in 10 mM phosphate buffer (pH 7.0) containing 0.5% hexa-decyltrimethylammonium bromide (HTAB) and were centrifuged for 20 min at 4°C at 20,000 ×*g*. MPO activity was measured in the supernatants (50 μl). An aliquot (50 μl) of the supernatant containing crude MPO was incubated at 37°C in an assay mixture with 0.1 mM H_2_O_2_ and 1.6 mM tetramethyl benzidine (TMB), followed by the measurement of optical density at 650 nm. One unit of the MPO activity is defined as the amount of enzyme required to degrade 1 μmol peroxide/ml, and is shown as unit/mg protein [18].

### Histopathological Study

To examine mucosal defects, hemorrhage, or inflammatory lesions, the isolated colons were fixed in 10%-buffered formalin, and embedded in blocks of paraffin. These colon-embedded paraffin blocks were cut into 5-μm thick sections, which were stained with hematoxylin-eosin, followed by examination using light microscopy.

### Immunoblot Analysis and ELISA

For the immunoblot analysis, the colon tissues were lysed with RIPA lysis buffer and centrifuged at 15,000 ×*g* at 4°C for 20 min. After extracting the colon lysates using SDS sample buffer, proteins were separated by 10% sodium dodecyl sulfate-polyacrylamide gel electrophoresis, and then electro-transferred onto nitrocellulose membrane. The membrane was blocked with 5% non-fat dried milk proteins, and probed with iNOS, COX-2, p65, p-p65, or β-actin antibodies. Each of these proteins was detected after incubations with a corresponding secondary antibody conjugated with horseradish peroxidase (HRP) for 1 h. Enhanced chemiluminescence (ECL) reagent was used to visualize the protein bands [18].

In the cytokine assay, the supernatants from the lysates of colon homogenates and peritoneal macrophages were added into 96-well ELISA plates. The expression levels of TNF-α, IL-1β, IL-6, and IL-10 were determined using ELISA kits for each according to the recommended protocol [18].

### Statistical Analysis 

All the results are shown as mean ± standard deviation (SD). Analysis of statistical significance was done using one-way analysis of variance (ANOVA) followed by post hoc analysis using Dunnett’s comparison tests. Differences at *p* < 0.05 were considered significant statistically.

## Results and Discussion

### L. Sakei S1 Inhibits the Pro-Inflammatory Cytokine Level in LPS-Stimulated Peritoneal Macrophages

**Fig. 1:**
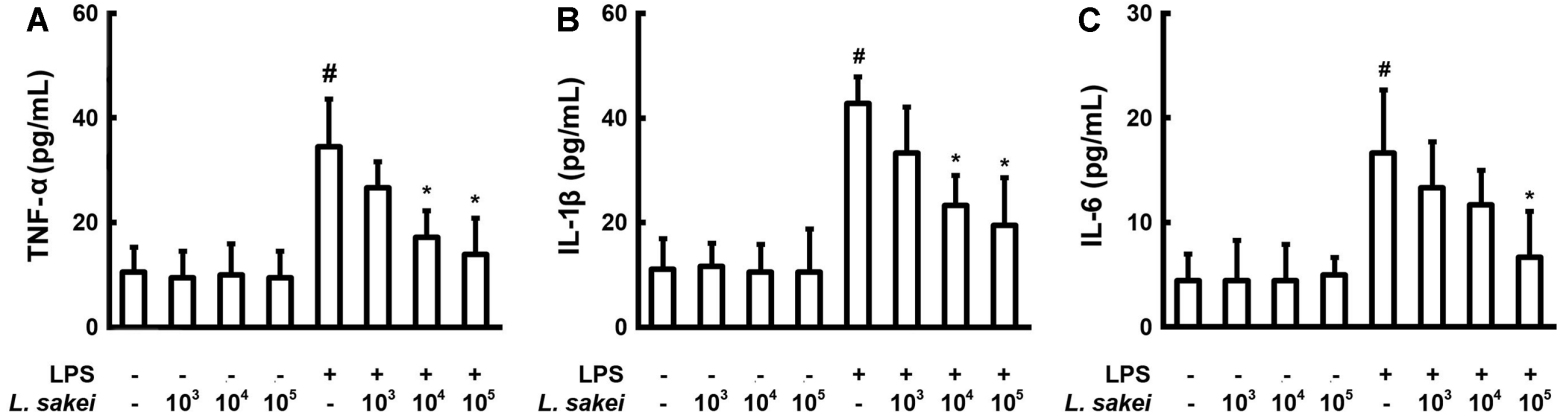
Effect of *Lactobacillus sakei* S1 on the expression of TNF-α (**A**), IL-1β (**B**), and IL-6 (**C**) in peritoneal macrophages, activated by LPS. 100 ng/ml LPS was used to treat the peritoneal macrophages (1 × 10^6^ cells/well) in the presence or absence of *L. sakei* S1 (1 × 10^3^, 1 × 10^4^, and 1 × 10^5^ CFU/well) for 20 h. ELISA was used to determine the levels of IL-1β, IL-6, and TNF-α in the culture supernatants. *L. sakei* S1 inactivated in boiling water bath for 30 min was employed. Enzyme activity values are represented as mean ± SD (*n* = 3). ^#^Significantly different in comparison with the normal control (*p* < 0.05). *Significantly different in comparison with the LPS alone-treated group (*p* < 0.05).

When the TNF-α inhibitory effect of 40 LAB strains (heat-treated) isolated from kimchi was measured in peritoneal macrophages stimulated with LPS, we discovered that S1 profoundly inhibited the synthesis of TNF-α. S1 also inhibited the elevated expression of IL-1β and IL-6 due to LPS induction (Fig. 1). These data are in agreement with the findings of Kwon *et al*., who showed that in peritoneal macrophages, blockade of IL-1β caused IL-6 inhibition through the interactions of IL-1β and IL-6 [19]. The expression of TNF-α, IL-1β, and IL-6 in S1-treated group (1 × 10^5^ CFU/ml) was significantly decreased by 59.7%, 54.5%, and 60%, respectively, in comparison with the LPS alone-treated control group. S1, which was selected as the most active strain, was identified as *L. sakei* through Gram staining and 16S ribosomal DNA sequencing.

### Resistance to Artificial Digestive Juices of L. Sakei S1 and Adhesion to Intestinal Epithelial Caco-2 Cells

In order to evaluate the possibility of using *L. sakei* S1 as a functional food material for humans, its resistance to artificial digestive juice and adhesion to Caco-2 cells were confirmed. Table 1 shows the viable counts of *L. sakei* S1, the strain isolated from kimchi after culturing in artificial digestion solution. *L. sakei* S1 was found to be resistant to strong acid at pH 2.5, with pepsin added at more than 3.0 ± 1.5 × 10^7^ CFU/ml. In order to confirm the resistance to artificial intestinal fluid, viable cell counts were checked, and more than 10^8^ CFU/ml of live cells were detected, so the resistance to artificial intestinal fluids was significantly high. These results are similar to those of the LAB used in currently available probiotic products [16]. Many LAB are known to exhibit a strong survival rate in the intestinal environment because of their high resistance to bile, which has been reported to be due to bile-enzyme [20].

On the other hand, the self-cohesive force indicates the clusterability between the same bacterial cells, and the self-cohesive force of probiotic strains correlates with the ability of the strain to adhere to intestinal epithelial cells. It is known that bacteria can easily form colonies in the intestinal tract and are resistant to this environment [21].

After incubation for 24 h, *L. sakei* S1 showed very high self-cohesion. In general, probiotic strains exhibit higher self-cohesion than that of pathogens, and strains with high adherence to hydrocarbons are known to have high self-cohesion [21]. In addition, some LAB have the ability to coagulate with the same strain or different species, thereby enhancing the adhesion to the epithelial cell mucosa. LAB are also able to bind with extracellular matrix molecules such as epithelial cells, mucosal layers, or mucosal components such as collagen, fibronectin, and vitronectin [22].

Adherence and proliferation of LAB on the surface of intestinal epithelial cells are important requirements for a probiotic strain. It is known that strains with strong adherence excel in metabolic and immunomodulating effects, induce immunological activity effectively, stabilize the intestinal mucosal barrier, and inhibit adherence of pathogenic bacteria to epithelial cells [22]. The resistance of *L. sakei* S1 to artificial digestive juice as well as its excellent adhesion to intestinal epithelial cells make this strain a promising material for functional foods.

### *L. sakei* S1 Ameliorates TNBS-Induced Colitis in Mice

Anti-colitic effects of *L. sakei* S1 were evaluated using a TNBS-induced colitis mouse model. The mechanisms of IBD pathogenesis in animals is generally studied using the experimental model of TNBS-induced colitis [23]. Inasmuch as the TNBS-induced colitis shows positive response to different currently available IBD therapies, such as anti-TNF alpha antibody treatment and sulfasalazine, this is a highly useful model to examine the efficacy of different novel anti-inflammatory therapies [24, 25].

**Fig. 2:**
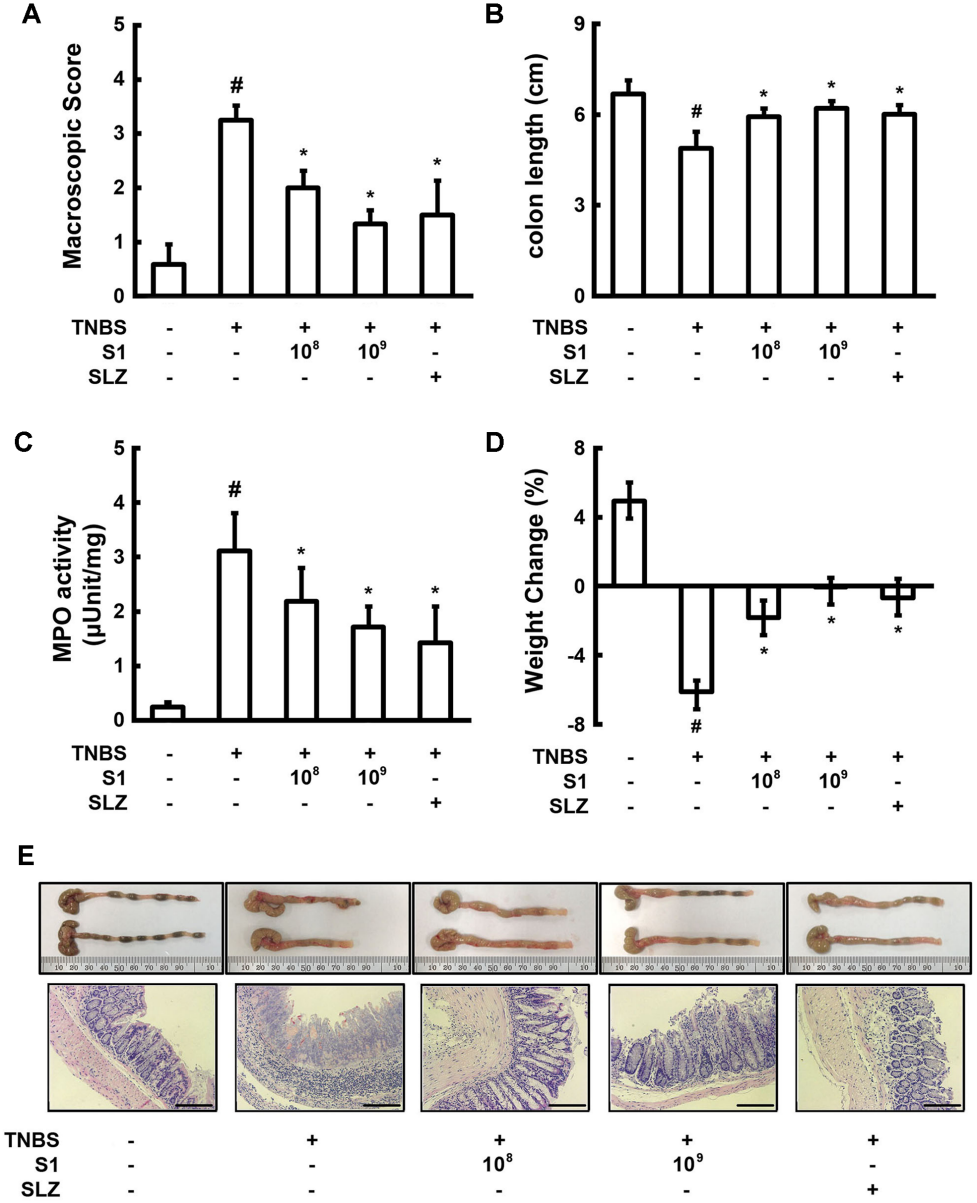
Effect of *Lactobacillus sakei* S1 on macroscopic disease (**A**), colon length (**B**), colonic MPO activity (**C**), body weight (**D**), and colon histology (**E**) in TNBS-induced colitis in mice. TNBS, except in the normal control group, was intrarectally administered to mice and test agents [saline, *L. sakei* S1 (1 × 10^8^ or 1 × 10^9^ CFU/ mouse), or sulfasalazine (SLZ; 50 mg/kg)] were orally administered for 3 days. The mice were anesthetized and sacrificed 20 h after the final treatment with LAB. All values are represented as mean ± SD (*n* = 6). ^#^Significantly different compared to the normal control group (*p* < 0.05). *Significantly different compared to the group treated with TNBS alone (*p* < 0.05). Scale bars: 50 μm.

Mice, following the intrathecal administration of TNBS, had loss of body weight, profound colonic inflammation and colon shortening (Fig. 2). The histological assessment of TNBS treated mouse colons revealed a high degree of bowel edema, intense infiltration of the mucosal superficial layers, in association with the destruction of the colonic epithelial cells. Administration of *L. sakei* S1 hindered weight loss, colon shortening, inflammation, and thickening of colons. *L. sakei* S1 also decreased bowel edema and inhibited infiltration of immune cells and destruction of epithelial cells in histological examination.

**Fig. 3:**
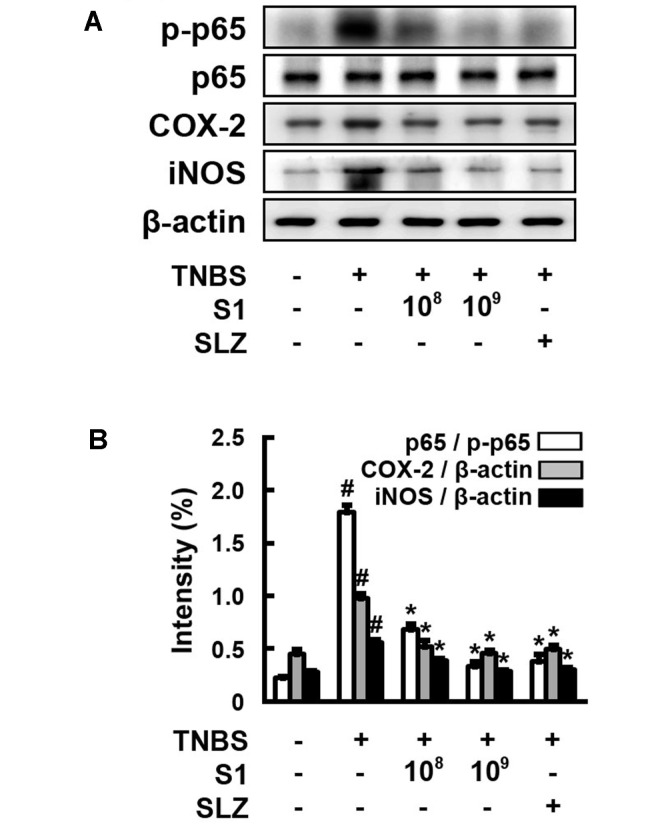
Effect of *Lactobacillus sakei* S1 on NF-κB in TNBSinduced colitis in mice. TNBS, except in the normal control group, was intrarectally administered to mice and test agents [saline, *L. sakei* S1 (1 × 10^8^ or 1 × 10^9^ CFU/mouse), or sulfasalazine (SLZ; 50 mg/kg)] were orally administered for 3 days. The mice were anesthetized and sacrificed 20 h after the final administration of *L. sakei* S1. The protein levels were determined by immunoblotting.

In addition, *L. sakei* S1 inhibited the TNBS-induced activity of MPO, a highly expressed enzyme in the granules that catalyzes the formation of highly reactive oxygen species (ROS), which are considered to be the biomarkers for oxidative damage. TNBS also promoted COX-2 and iNOS expression and activation of NF-κB (Fig. 3). Enhanced production of COX-2 and iNOS has been described in IBD and also in the animal models of intestinal inflammation [19]. *L. sakei* S1 inhibited the induction of p65 phosphorylation by TNBS and decreased the TNBS-induced expression of iNOS and COX-2. The inhibitory effect of *L. sakei* S1 (1 × 10^9^ CFU) was more than that of sulfasalazine (50 mg/kg).

**Fig. 4:**
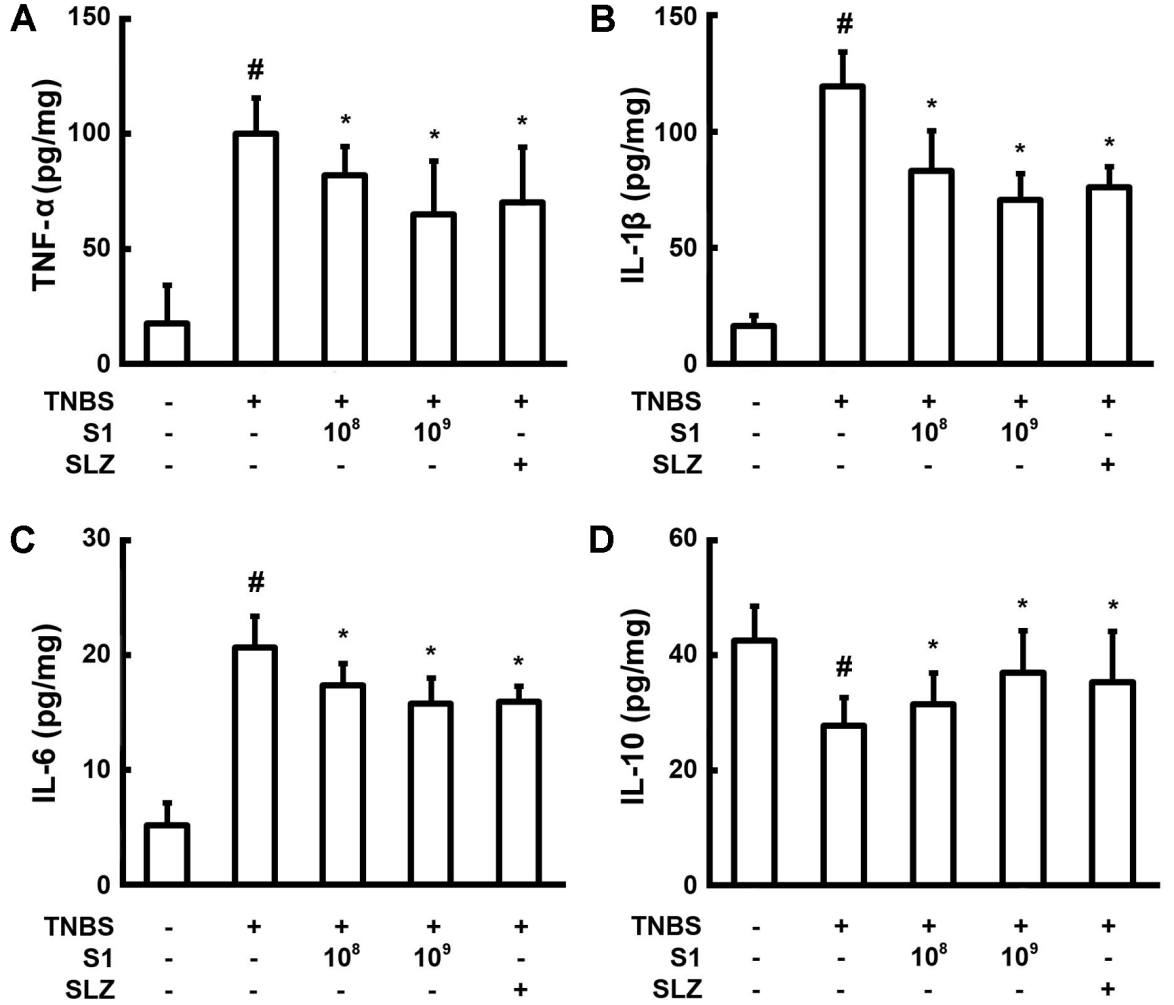
The effect of *Lactobacillus sakei* S1 on the synthesis of pro-inflammatory cytokines TNF-α (**A**), IL-1β (**B**), and IL-6 (**C**), and anti-inflammatory cytokine IL-10 (**D**) in TNBS-induced colitis in mice. TNBS, except in the normal control group, was intrarectally administered to mice and test agents [saline, *L. sakei* S1 (1 × 10^8^ or 1 × 10^9^ CFU/mouse), or sulfasalazine (SLZ; 50 mg/kg)] were orally administered for 3 days. The mice were anesthetized and sacrificed 20 h after the final administration of *L. sakei* S1. The cytokine levels in colon were measured by using ELISA. All values are represented as mean ± SD (*n* = 6). ^#^Significantly different compared to the normal control group (*p* < 0.05). *Significantly different compared to the group treated with TNBS alone (*p* < 0.05).

When pro-inflammatory cytokine levels were measured in colon lysate, TNBS increased cytokine levels, including TNF-α, IL-1β, and IL-6 but decreased the expression of IL-10 (Fig. 4). Oral administration of *L. sakei* S1 reduced the expression of TNF-α, IL-1β, and IL-6. However, *L. sakei* S1 strongly increased the expression of IL-10. The cytokine IL-10 has potent anti-inflammatory properties and thereby curtails immune responses of the host to pathogens. Thus, IL-10 alleviates injury to the host and at the same time maintains normal tissue homeostasis [26]. Dysregulation of IL-10 function and synthesis is accompanied by aggravated immunopathological responses towards infection and also elevated risk for IBD and many such inflammatory diseases [26]. *L. sakei* S1 treatment recovered 33.2% of the IL-10 expression compared to the negative control, whereas it inhibited TNF-α, IL-1β, and IL-6 cytokine expression by 35%, 41%, and 23.6%, respectively.

Many normal cellular functions such as proliferation, adhesion and inflammatory responses are controlled by the ubiquitous transcription factor, NF-κB [6]. NF-κB, which is normally present as an inactive heterodimer of p50 and p65 (RelA) subunits in the cytoplasm, translocates to the nucleus following stimulation by endotoxins such as LPS. The activated NF-κB (pp65) in the nucleus, binds its specific sites on DNA, and triggers the transcriptional activation for the expression of TNF-α and IL-1β [27]. These pro-inflammatory cytokines further activate NF-κB through a positive feedback mechanism [28]. Thus, it is necessary to block these inflammatory cytokines as much as possible to inhibit NF-κB signaling in IBD. For these reasons, anti-TNF-α antibodies have been used clinically. However, it is very important to develop safe therapeutic LAB formulations, because the reactivity of drugs used in clinical practice is low, and side effect problems remain [29]. *L. sakei* S1 suppressed inflammatory cytokines while inhibiting NF-κB, and it may be developed as a functional LAB for colitis.

Although inflammation is a protective response of the host immunity, abnormal inflammatory responses can cause host tissue damage [30]. IBD is a severe inflammatory disease of the intestine in humans, and it is essential to clearly understand the pathogenesis of IBD, because its incidence is increasing steadily worldwide [30]. The pathogenesis of IBD is considered to be mediated through a complex intestinal mucosal immune response towards the resident intestinal microflora [31]. This study showed that endotoxins such as LPS produced by gut microbiota stimulated the NF-κB pathway, which might be a possible cause of IBD. *L. sakei* showed the possibility of ameliorating colitis by inhibiting the NF-κB pathway.

Collectively, the above findings suggest that *L. sakei* S1 likely prevents colitis by antagonizing the NF-κB pathway and thereby inhibiting the formation of TNF-α, IL-1β, and IL-6, the major pro-inflammatory cytokines. In addition, *L. sakei* S1, a plant-derived LAB isolated from kimchi, a representative fermented food in Korea, may be a material for functional food applications against IBD.

## Figures and Tables

**Table 1:** Resistance to artificial digestive juices of *Lactobacillus sakei* S1 and adhesion to intestinal epithelial Caco-2 cells.

	Viable cell counts (CFU/ml)			Auto aggregation (%)

	Gastric juice	Intestinal juice	Adhesion	
*L. sakei* S1	3.0 ± 1.5 × 10^7^	1.6 ± 0.5 × 10^8^	4.0 ± 1.1× 10^5^	45.6 ± 5.5

Numbers of *L. sakei* S1 were counted by plating serial dilutions (in diluted anaerobic broth, pH 7.2) on MRS and BL agars followed by anaerobic incubation at 37°C for 48 h. All values are mean ± SD (*n* = 6).
